# A twisted tale of limbs: fibular aplasia with tibial campomelia and oligosyndactyly

**DOI:** 10.11604/pamj.2026.54.20.49804

**Published:** 2026-05-25

**Authors:** Gahana Kataria, Vaishali Dhawan

**Affiliations:** 1 Department of Radio-Diagnosis, Jawaharlal Nehru Medical College, Datta Meghe Institute of Higher Education and Research (DU), Sawangi (Meghe), Wardha, Maharashtra, India

**Keywords:** Fibular aplasia, tibial campomelia, oligosyndactyly, FATCO syndrome, congenital limb deformity

## Image in medicine

A 3-year-old female child was evaluated for deformity and shortening of the right lower limb, which had been evident since birth. No maternal history of drug intake during pregnancy; however, the mother was a technician working in a radiation lab. On clinical examination, the affected leg appeared bowed, with fewer and smaller toes than on the opposite side. Radiographic assessment revealed a complete absence of the right fibula, along with pronounced anterior curvature of the tibia (tibial campomelia) and reduced digital count (oligosyndactyly). The contralateral limb was normal. These findings were consistent with the fibular aplasia-tibial campomelia-oligosyndactyly (FATCO) spectrum, an uncommon congenital anomaly involving defective mesenchymal development of the lower limb. It is a syndrome of unknown genetic basis and inheritance with variable expressivity and penetrance. Orthopaedic management included plans for gradual limb lengthening, with amputation and prosthetic fitting considered to enhance function and mobility.

**Figure 1:**
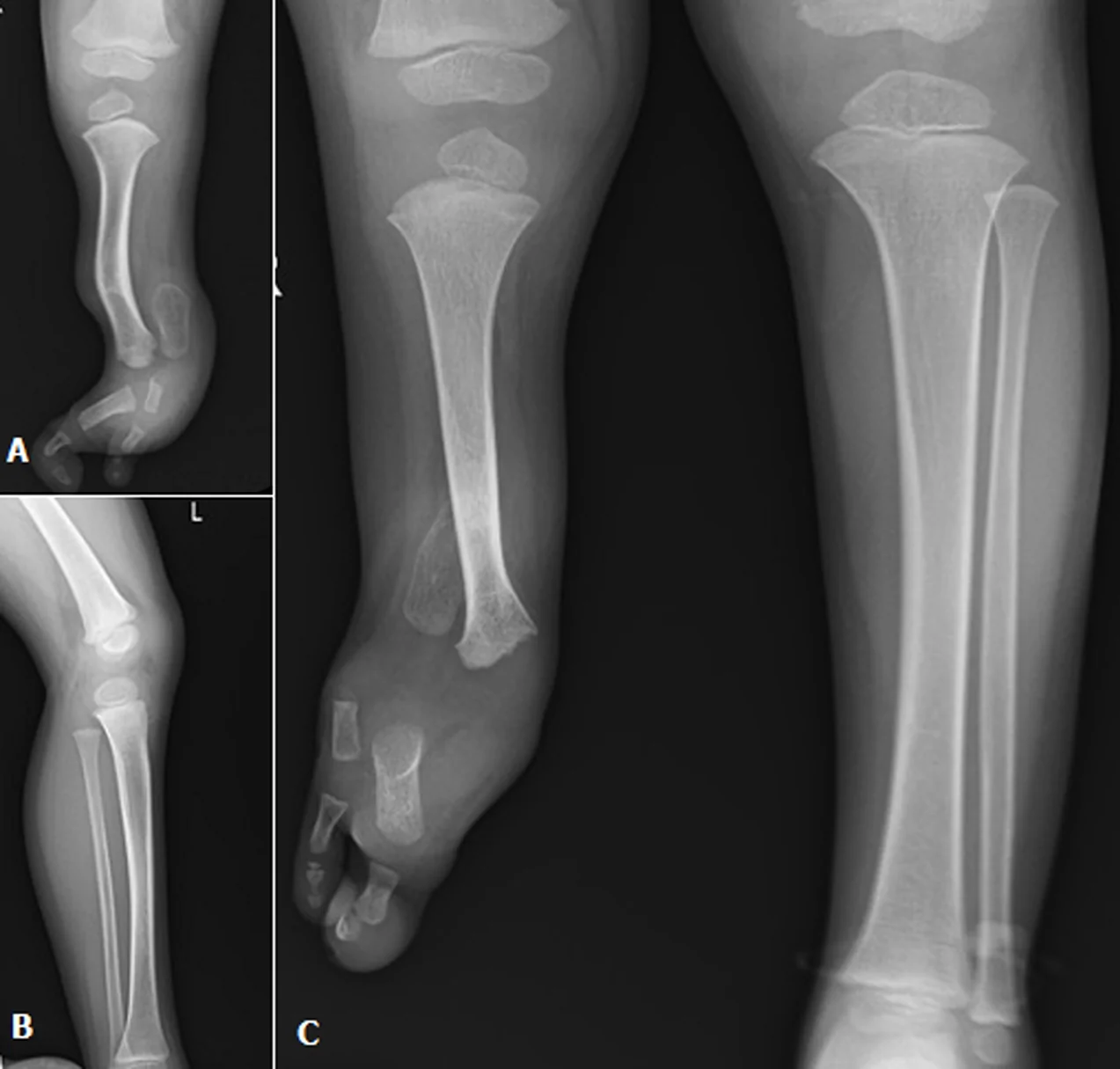
radiographs of the lower limbs showing: A, B) anteroposterior and lateral views of the right leg demonstrating complete absence of fibula, anterior bowing of the tibia, and oligosyndactyly of the right foot; C) comparative view of both lower limbs showing a normal left leg and shortened, deformed right leg consistent with FATCO syndrome

